# Re-weighting of Sound Localization Cues by Audiovisual Training

**DOI:** 10.3389/fnins.2019.01164

**Published:** 2019-11-15

**Authors:** Daniel P. Kumpik, Connor Campbell, Jan W. H. Schnupp, Andrew J. King

**Affiliations:** Department of Physiology, Anatomy and Genetics, University of Oxford, Oxford, United Kingdom

**Keywords:** auditory-visual perception, sound localization, binaural, head-related transfer function, ventriloquism aftereffect, cue integration, multisensory, behavioral training

## Abstract

Sound localization requires the integration in the brain of auditory spatial cues generated by interactions with the external ears, head and body. Perceptual learning studies have shown that the relative weighting of these cues can change in a context-dependent fashion if their relative reliability is altered. One factor that may influence this process is vision, which tends to dominate localization judgments when both modalities are present and induces a recalibration of auditory space if they become misaligned. It is not known, however, whether vision can alter the weighting of individual auditory localization cues. Using virtual acoustic space stimuli, we measured changes in subjects’ sound localization biases and binaural localization cue weights after ∼50 min of training on audiovisual tasks in which visual stimuli were either informative or not about the location of broadband sounds. Four different spatial configurations were used in which we varied the relative reliability of the binaural cues: interaural time differences (ITDs) and frequency-dependent interaural level differences (ILDs). In most subjects and experiments, ILDs were weighted more highly than ITDs before training. When visual cues were spatially uninformative, some subjects showed a reduction in auditory localization bias and the relative weighting of ILDs increased after training with congruent binaural cues. ILDs were also upweighted if they were paired with spatially-congruent visual cues, and the largest group-level improvements in sound localization accuracy occurred when both binaural cues were matched to visual stimuli. These data suggest that binaural cue reweighting reflects baseline differences in the relative weights of ILDs and ITDs, but is also shaped by the availability of congruent visual stimuli. Training subjects with consistently misaligned binaural and visual cues produced the ventriloquism aftereffect, i.e., a corresponding shift in auditory localization bias, without affecting the inter-subject variability in sound localization judgments or their binaural cue weights. Our results show that the relative weighting of different auditory localization cues can be changed by training in ways that depend on their reliability as well as the availability of visual spatial information, with the largest improvements in sound localization likely to result from training with fully congruent audiovisual information.

## Introduction

Accurate sound localization is achieved by integrating binaural cues (interaural level and time differences; ILDs and ITDs) and location-dependent spectral cues, which together constitute the head-related transfer function (HRTF). ITDs and ILDs provide information about a sound’s azimuth, whereas spectral cues are crucial for sound elevation judgments and for resolving front-back confusions (Wightman and Kistler, 1993; Jin et al., 2004). Depending on the frequency content of the sound and the egocentric location of its source, each of these cues alone may be spatially ambiguous, leading to localization errors (Blauert, 1997; Shinn-Cunningham et al., 2000; King et al., 2001). To resolve ambiguities between multiple redundant cues and form a final estimate of sound location, the brain integrates the cues by giving greater weight to those cues that are more reliable (Reijniers et al., 2014), with “reliability” defined as the spatial precision of a cue (Trommershäuser et al., 2011).

Studies in which binaural cues are altered by temporarily occluding one ear (Kacelnik et al., 2006; Van Wanrooij and Van Opstal, 2007; Kumpik et al., 2010; Keating et al., 2013, 2016) demonstrate that localization cue weights can change with experience. The auditory system can adapt rapidly to distortions in spatial hearing by giving greater weight for azimuthal localization to the cues that are less affected by the perturbation, i.e., the unchanged spectral cues provided by the non-occluded ear. Recent work in ferrets (Keating et al., 2013, 2015, 2016) and humans (Keating et al., 2016) has shown that such adaptation can be achieved either by up-weighting these cues or by learning a new relationship between the altered binaural cues and directions in space, depending on the spectral content of the stimuli used and therefore the localization cues that are available.

The importance of cue reliability for integration and remapping of sensory inputs extends across sensory modalities, with dramatic demonstrations of the dominance of vision over audition in spatial judgment tasks. For example, in the presence of a spatially displaced visual distractor, judgments of sound location are biased in the direction of the visual stimulus (the ventriloquism effect; Bertelson and Radeau, 1981). Similarly, using lenses to compress the visual field results in a corresponding change in the perception of auditory space (Zwiers et al., 2003). The ventriloquism illusion has been shown to reflect the greater spatial reliability of one sensory modality (vision) over the other (audition), with the opposite effect – audition dominating audiovisual location judgments – occurring when visual information becomes spatially blurred and so less reliable (Alais and Burr, 2004). Moreover, after exposure to spatially-discordant auditory and visual stimuli, subjects often show a shift of auditory localization in the direction of the previously presented visual stimulus (the ventriloquism aftereffect, VAE; Radeau and Bertelson, 1974; Recanzone, 1998). This remapping of auditory space can be characterized as an updating of auditory likelihoods following exposure to spatially-conflicting but more reliable visual information (Wozny and Shams, 2011a).

Further evidence that cue reliability may determine how multisensory spatial information is integrated is provided by the demonstration that co-located visual cues can both improve the accuracy of sound localization judgments (Bolognini et al., 2007; Tabry et al., 2013; Hammond-Kenny et al., 2017) and contribute to the suppression of echoes (Bishop et al., 2011). Adaptive changes in sound localization accuracy following manipulation of auditory spatial cues can take place, however, in the absence of visual feedback (Kacelnik et al., 2006; Carlile and Blackman, 2014), suggesting that the auditory system may be able to take advantage of other contextually reliable information, such as sensorimotor feedback from head movements, or possibly even other sound localization cues, to recalibrate representations of space after normal hearing is perturbed. Nevertheless, training paradigms that include visual cues can facilitate the ability of listeners to learn new associations between auditory spatial cues and directions in space (Strelnikov et al., 2011; Berger et al., 2018) and to utilize ILDs appropriately following bilateral cochlear implantation (Isaiah et al., 2014).

Together, these studies show that vision can have a profound impact on auditory localization, most likely due to its greater spatial reliability. Little is known, however, about the influence of visual inputs on behavioral sensitivity to different auditory spatial cues (Sarlat et al., 2006). Given that the perceptual weights of monaural and binaural cues can change following unilateral hearing loss, we performed four short-term training experiments that used different configurations of audiovisual spatial congruence to test the hypothesis that binaural cue re-weighting occurs when the trial-to-trial spatial accuracy of each cue is signaled by its spatial congruence or incongruence with a visual stimulus. To directly assess the role of visual information, a second group of subjects performed a training task that directed visual attention in a similar fashion, and used the same auditory spatial configurations, but did not provide spatially-relevant visual cues.

We used an ILD/ITD cue-trading paradigm to assess how sound localization biases and cue weights changed after training. Our overall hypothesis was that training with spatially-informative visual cues (the AV+ group) would lead to larger changes in auditory localization performance than when the timing and location of the visual stimulus on each trial was unrelated to that of the sound (the AV− group). More specifically, since brief exposure to audiovisual stimuli has been shown to improve auditory localization with non-individualized HRTFs (Berger et al., 2018), we expected to see reductions in localization bias when ITDs and ILDs were both spatially congruent with the visual stimuli (AV+, natural-0) and that these bias reductions would be larger than if the value of one of those cues was randomized from trial to trial (AV+, random ITD; AV+, random-ILD). Furthermore, in line with our general hypothesis regarding the importance of contextual reliability for cue re-weighting, we anticipated that the binaural cue that contributed more to sound localization prior to training would be further up-weighted, and that this effect would be stronger for the AV+ group than the AV− group. On the other hand, reducing cue reliability by randomizing either the ITD or ILD should result in less upweighting of that cue. Finally, we included a task in which visual stimuli were offset from the corresponding auditory cue value by 10° (AV+, natural-10). We hypothesized that training subjects on this task would result in strong remapping of sound localization biases (the ventriloquism aftereffect), but not when the same auditory cue values were presented in the absence of spatially-informative visual signals (AV−, natural-10), and that remapping would be associated with less cue reweighting than after training with spatially-congruent audiovisual stimuli.

Our results show that changes in auditory localization biases and cue weights can occur both in the presence and absence of a visual teacher signal, with the largest changes in bias, and presumably localization accuracy, occurring when spatially-informative visual cues were provided during training. While adjustments in perceptual cue weights were also affected by the availability of visual information, this additionally appears to depend on the relative reliability of the cues given the current acoustic environment. Consequently, the expected change in weights was observed when ITDs were randomized, but not when ILDs were randomized. These findings therefore provide insight into the changes in auditory processing that occur when auditory cues are spatially matched or mismatched with vision, and support the notion that the brain can integrate different cues for sound source location in a highly flexible fashion.

## Materials and Methods

### Subjects

Thirty two subjects took part in the experiment (14 male, mean age ± SD: 23.6 ± 3.7 years), 20 of whom were randomly assigned to perform audiovisual training with spatially-informative visual cues (the AV+ group). The rest carried out audiovisual training with visual cues that were spatially uninformative (AV−). All had normal or corrected-to-normal vision and normal audiometric thresholds (≤20 dB HL) from 125 Hz to 8 kHz. The different audiovisual experiments were run in random order and were completed on different days. Subjects were recruited through online and departmental notices; all received payment for their time and provided informed consent before beginning the study. Ethical approval was provided by the Medical Sciences Inter-Divisional Research Ethics Committee of the University of Oxford (study R52936).

### Apparatus

Subjects sat on a stool in a sound-attenuated chamber and a chin-rest was used to keep their head stationary during the experiment. Virtual auditory space (VAS) stimuli were passed to a MOTU 828 MKII audio interface and presented to subjects over Sennheiser HD650 headphones. A Viewsonic PJD5453S projector, with a maximum resolution of 1920 × 1080 pixels, was mounted on the wall behind and above the subject and projected stimuli onto a semicircular screen that curved around the subject and covered azimuths up to 70° to the left and right of the midline. The screen was positioned at a radius of 84 cm from the center of the subjects’ heads, and extended from 88 to 144 cm above the floor (thus covering 36.9° of visual field from top to bottom). It was composed of black speaker cloth mounted on a wooden frame, and a black cotton curtain was attached with velcro to the bottom of the frame to hide the metal legs. A computer mouse was provided for subjects to move a crosshair cursor that was projected onto the screen in order to initiate and respond to trials. During the experiments, the azimuthal location of the visual and auditory stimuli was varied as described in further detail in the following section, while the vertical location (elevation) was held fixed at 116 cm above the floor, corresponding roughly to eye level.

### Stimuli

#### Auditory Stimuli

Broadband Gaussian noise pulses were generated on each trial and were bandpass filtered and convolved with appropriate spatial cues according to the type of psychophysics run being conducted (see below). All auditory stimuli were presented over headphones calibrated to an RMS average binaural level of 70 dB SPL, using a Brüel and Kjær 4191 condenser microphone placed inside a Brüel and Kjær 4153 artificial ear and connected to a Brüel and Kjær 3110-003 measuring amplifier. One hundred percent sinusoidal amplitude modulation (SAM) was applied at a rate of 6 Hz; in the sound localization and cue trading tasks, stimuli spanned 8 cycles of the modulator and were therefore 1.33 s in duration. In the spatial flash detection and oddball tasks used for audiovisual training, between 6 and 12 noise pulses were presented per trial with an inter-pulse interval of 200 ms; each noise pulse was a single cycle of the modulator and therefore had a duration of 166.7 ms, and the duration of the full stimulus sequence was 4.2 s (or shorter if subjects responded before the end).

Although non-individualized HRTFs selected from the SYMARE database (Jin et al., 2014) were used, we applied reverberations using a custom-designed room simulator, which added specular reflections of up to third-order to the presented sounds, in order to generate realistically externalized auditory stimuli and thus encourage multisensory integration. The SYMARE database comprises head-related impulse response filter measurements for 393 source directions, sampling auditory space in ≤10° intervals from 45° below the horizon. We psychophysically determined which of the 10 individual HRTFs available in the database was best for each subject before training them on a sound localization task with the chosen HRTF (see section Procedure). The ITDs for each virtual direction were applied as required for each experimental condition. We were thus able to manipulate the frequency-dependent ILDs, which incorporate the spectral cues at each ear, independently from the ITD cue.

We combined sound localization cues in three general configurations that were used for different phases of the study. The first configuration was for HRTF selection, ILD-only localization training and ITD-only localization training; to facilitate learning for each cue, we attempted to isolate the effects of ILDs and ITDs by providing training on each with the other cue set to zero. ILD-only localization stimuli were used in the first (familiarization) session to select an appropriate HRTF for each subject, and then to train subjects with the ILDs from that HRTF. The periods between experimental sessions, in which subjects obviously localized sounds using their own ears, varied in duration. In some cases, this appeared to result in inflated variability in ILD localization responses with the non-individualized HRTF early in the next session. We therefore also used ILD-only stimuli to re-familiarize subjects with the ILD cues before each audiovisual training experiment (see section Procedure and Table 1). ITD-only stimuli were only used for localization training purposes. ITDs are spatially ambiguous for high-frequency stimuli, whereas free-field ILDs (and spectral cues) tend to be small, though still usable, for low-frequency stimuli (Hartmann et al., 2016), a dichotomy known as the Duplex theory of sound localization (Strutt, 1907). The ILD stimuli were therefore band-pass filtered from 1.9 to 16 kHz and the ITD stimuli were band-passed from 0.5 to 1.3 kHz, in both cases to avoid frequency regions in which the other cue (which had a value corresponding to 0°) could play a prominent role in localization. To ensure that training was confined to the basic localization cues, reverberation was not added to these stimuli.

**TABLE 1:** Stimulus parameters for sound localization tasks.

**Session number**	**Experimental phase**	**Localization cue(s)**	**Post-trial visual feedback**	**Stimulus bandwidth**	**Zeroed ILD**	**Zeroed ITD**	**Task**
1	HRTF selection	ILDs	Yes	1.9–16 kHz	–	✓	Procedural familiarization
		ILDs with each HRTF in turn	No	1.9–16 kHz	–	✓	HRTF selection
	Auditory localization training	ILDs	Yes	1.9–16 kHz	–	✓	ILD training with selected HRTF
		ITDs	No	0.5–1.3 kHz	✓	–	ITD familiarization

2–5 (first hour)	Matching ILD and ITD angles	ILDs	Yes	1.9–16 kHz	–	✓	Procedural and ILD familiarization
		ILDs	No	1.9–16 kHz	–	✓	Unsupervised performance with ILDs
		ITDs	No	0.5–1.3 kHz	✓	–	Match ITDs to ILD angles
		Natural (congruent ILDs and ITDs)	No	0.5–16 kHz	–	–	Sound localization with matched ILDs and ITDs
2–5 (second hour: before and after audiospatial recalibration)	Pre/post audiospatial recalibration tests	All ILD and ITD combinations	No	0.5–16 kHz	–	–	Sound localization (cue trading)

The second stimulus configuration used ILDs and ITDs that were always congruent, while the third stimulus configuration employed ILDs and ITDs that were in spatial conflict. In both cases, broadband noise (0.5–16 kHz) was used as the stimulus, and reverberation was applied to the impulse response functions and was thus temporally congruent with the ITD cue.

#### Visual Stimuli

All visual stimuli and markers were white on a black background. Geometric correction was applied to take account of the curvature of the screen. We used three types of visual stimuli: (1) a 0.48° wide fixation dot, which was located in front of the subject at 0° azimuth, and at 13.4° elevation to displace it from the locations to be used for visual feedback during training; (2) a 1.23° wide cross hair mouse cursor, which subjects could control to initiate trials by clicking on the fixation dot, or respond on each trial by clicking on the location associated with the perceived sound-source direction; (3) during both pre-experimental sound localization training and the audiospatial recalibration experiments, we presented visual stimuli at specific angles relative to the horizontal position of the sound source in the form of blobs with a Gaussian contrast profile that covered 0.61° of visual field at full width half-maximum. Visual feedback stimuli were always presented at 0° elevation.

### Psychophysical Tasks

Psychophysical tasks were controlled using Matlab (r.2015b; Mathworks) and its associated Psychophysics Toolbox (Brainard, 1997; Pelli, 1997; Kleiner et al., 2007).

#### Sound Localization Task

**FIGURE 1:**
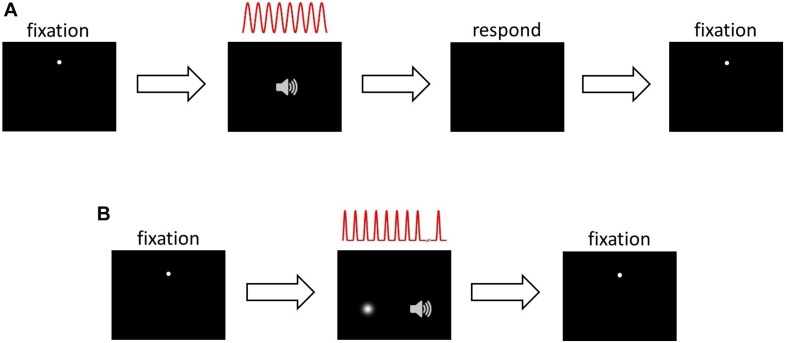
Psychophysical task structure. **(A)** Sound localization. Panel 1, inter-trial interval: subject fixates on white dot, moves mouse cursor over it and left clicks to initiate trial. Panel 2, stimulus presentation: 200 ms after trial onset, 8 cycles of a sinusoidally amplitude modulated (SAM) auditory noise stimulus were presented, depicted in red. The auditory stimulus was presented in virtual acoustic space incorporating ILDs, ITDs or both cues. Panel 3, response: after stimulus presentation, subject makes an eye movement in the perceived direction of the auditory stimulus, then moves the mouse cursor over that position and left clicks to respond. Panel 4, inter-trial interval and start of next trial. **(B)** Audiospatial recalibration. Panel 1, as in **(A)**. Panel 2, audiovisual stimulus presentation and either visual flash detection (AV− group) or visual spatial oddball detection (AV+ group): 200 ms after trial onset, a sequence of up to 12 auditory SAM cycles, separated by 200 ms, was presented. Subjects were required to ignore the auditory stimuli and monitor the visual scene: before each trial the AV− group were visually cued to attend a random location, which they monitored for a brief, spatiotemporally random flash. The AV+ group were presented with a visual “teacher” stimulus sequence during each trial, which they monitored for a spatial “oddball” that occurred between the 6th and the 11th (inclusive) stimulus in the sequence. The visual sequence in the AV+ group was temporally congruent with the auditory stimulus sequence. Subjects were required to left click when they detected the flash (AV−) or oddball (AV+). At this point stimulus presentation was halted. Panel 3, inter-trial interval and start of next trial.

This task (Figure 1A) was used during HRTF selection, ILD and ITD training, and during the pre- and post-audiovisual training test blocks, with the session type determining the sound localization cues included in the stimuli and whether or not visual error feedback was provided (see section Procedure). Subjects were seated in the chamber at the center of the semicircular screen and fitted with headphones. They initiated trials by clicking the cross hair mouse cursor on the fixation dot. After 200 ms, the fixation dot disappeared and an auditory stimulus was presented from the selected virtual location. Subjects were instructed to direct their gaze in the perceived direction of the sound source, and then respond by moving the cross hair cursor to that location and clicking the mouse button. Once the subject had responded, the fixation dot reappeared in preparation for the next trial.

#### Audiospatial Recalibration With Spatially-Informative Visual Cues (AV+) (Spatial Oddball Detection) Task

We used an audiovisual oddball detection task (Figure 1B) where the position of the visual stimulus acted as a potential “teacher” signal for recalibration of auditory space. Trials were initiated in the same way as the localization task. Two hundred milliseconds after the fixation dot and mouse cursor had disappeared, a sequence of auditory stimuli was presented, each consisting of a single modulation cycle (166.7 ms duration and separated by 200 ms) of the SAM noise stimulus. Concurrently with each auditory stimulus, a visual “Gaussian blob” stimulus (i.e., a bright patch with brightness fading at the edges according to a 2-D Gaussian spatial luminance profile) was presented, with the blob’s contrast rising and falling in synchrony with the amplitude modulation of the auditory stimulus.

Up to 12 temporally concurrent auditory and visual stimuli were presented in the sequence. Eleven of the visual stimuli were presented at a single standard location in each trial (−20°, −10°, 0°, 10°, or 20° azimuth; negative angles represent stimuli to the left of the midline), but one was a spatial oddball that could randomly appear as the 6th to the 11th stimulus in the sequence. The oddball was offset by either 10° or 20° to the left or right of the standard stimulus location. Subjects were asked to ignore the sounds and to direct their gaze to the standard visual stimulus sequence, then respond by clicking the mouse button when they detected the oddball. At this point, stimulus presentation was terminated, the fixation dot returned and the next trial was ready to be initiated. On each audiovisual presentation during the stimulus sequence, the spatial relationship between the ILD and ITD cues and the visual stimuli was manipulated in different ways, depending on the training experiment (see section Procedure and Table 2). Each of the possible combinations for the standard and oddball angles (5 standard angles × 2 oddball angles × 2 oddball directions relative to standard) was presented once, giving 20 trials per audiovisual training block. There were 20 blocks during the audiovisual training phase of each experiment, with subjects receiving a 30 s break between blocks. Subjects remained in the chamber with the door closed for the duration of the audiovisual training phase (∼50 min) and subsequent post-training localization test (∼5 min), and were asked to keep the headphones on during this period.

**TABLE 2:** Stimulus parameters for cue re-weighting (audiospatial recalibration) phase.

**Session number**	**Visual configuration**	**Task**	**Experiment**	**Binaural configuration**			
				**Distribution across midline**	**Intra-trial ILD position**	**Intra-trial ITD position**	**ILDs and ITDs spatially congruent**
S2–S5 (second hour)	Spatially and temporally unrelated to auditory stimuli (AV−)	Visual flash detection	Natural-0	Symmetric	Constant	Constant	✓
			Random-ITD	Symmetric	Constant	Random	x
			Random-ILD	Symmetric	Random	Constant	x
			Natural-10	Asymmetric	Constant	Constant	✓
	
	Temporally matched to auditory stimuli and spatially informative (AV+)	Visual oddball detection	Natural-0	Symmetric	Constant	Constant	✓
			Random-ITD	Symmetric	Constant	Random	x
			Random-ILD	Symmetric	Random	Constant	x
			Natural-10	Asymmetric	Constant	Constant	✓

*When the intra-trial ILD and ITD positions were “constant” within a stimulus sequence (columns 6 and 7), this excludes a spatial oddball.*

#### Audiospatial Recalibration With Spatially-Uninformative Visual Cues (AV−) (Flash Detection Task)

To remove the influence of spatiotemporally concurrent visual stimulation while ensuring that visual attention was engaged in the same way as in the AV+ group, subjects in the AV− group performed an identical task with two important differences. First, after triggering a trial but before the start of each auditory stimulus sequence, subjects were presented with a Gaussian blob for 1 s to cue their attention to a random location between −20° and 20° from the midline. Second, during the auditory sequence no spatiotemporally congruent visual stimuli were presented. However, at a random onset time between the onset of the 6th and the offset of the 11th stimulus in the auditory sequence, a Gaussian blob (33.3 ms) was presented up to 20° away from the cued visual location. Subjects were instructed to fixate on the cued location and click the mouse as soon as possible after the presentation of the brief flash. The flash was never spatiotemporally congruent with the sounds. Subjects were told that reaction time was being measured.

For both the AV+ and AV− tasks, the mean (± SD) number of stimulus pulses presented during each trial sequence was 8.5 ± 0.4 across all subjects and conditions, and the mean interval between sessions was 6.09 ± 12.34 days.

### Procedure

#### Session 1

##### HRTF selection phase (∼1 h)

The first 2 h session of the experiment involved two phases, HRTF selection and auditory-only localization training. It was first necessary to determine, for each subject, which HRTF produced the most veridical sound localization performance. Subjects therefore completed:

(i) A single ILD-only localization run with ITDs fixed at 0 μs, using a randomly chosen HRTF and high-pass noise (1.9–16 kHz), to familiarize them with the setup and the localization task. ILDs corresponding to stimulus angles of −20°, −10°, 0°, 10,° and 20° (15 repetitions/angle) were used and visual feedback to stimulus location was given for a period of 400 ms after each trial.
(ii) HRTF selection runs (high-pass noise, 1.9–16 kHz) with each HRTF tested in random order, ITDs fixed at 0 μs. Stimulus angles were −20°, −10°, 0°, 10°, and 20° (10 repetitions/angle) and no visual feedback was provided. Sound localization performance on this task identified which HRTF provided the most veridical ILD cues, as determined by the HRTF that produced a slope closest to 1°/° when response angle was regressed on virtual stimulus angle.

##### Auditory localization training phase (∼1 h)

Once the most appropriate HRTF had been selected, the first session concluded with a sound localization training phase, designed to familiarize subjects with the chosen non-individualized ILDs. They also performed an ITD localization task during this phase to provide exposure to the range of ITDs presented over headphones. Subjects completed:

(i) ILD-only training with visual feedback after each trial, using high-pass noise (1.9–16 kHz). Stimulus angles of −20°, −10°, 0°, 10°, and 20° were used (15 repetitions/angle).
(ii) Familiarization with ITD-only localization using band-pass noise (0.5–1.3 kHz). We used ITDs up to 145.8 μs on either side of the midline in 21.8 μs steps (5 repetitions/ITD). Because of the consistent relationship between ITDs and frontal sound locations, no visual feedback was provided in these runs, but subjects were informed that the stimuli could now come from anywhere within this range. The task was run repeatedly to reduce the mean square error (MSE) of the fit when response angle was regressed on ITD.

#### Sessions 2–5

##### Matching ITDs to virtual sound directions (∼1 h)

In the first hour of each of the following 2 h experimental audiospatial recalibration sessions (AV− or AV+), subjects first performed localization tasks that were intended to re-familiarize them with the auditory stimuli and also to determine a set of ITDs to match the ILD angles to be used in the experimental training block.

**FIGURE 2:**
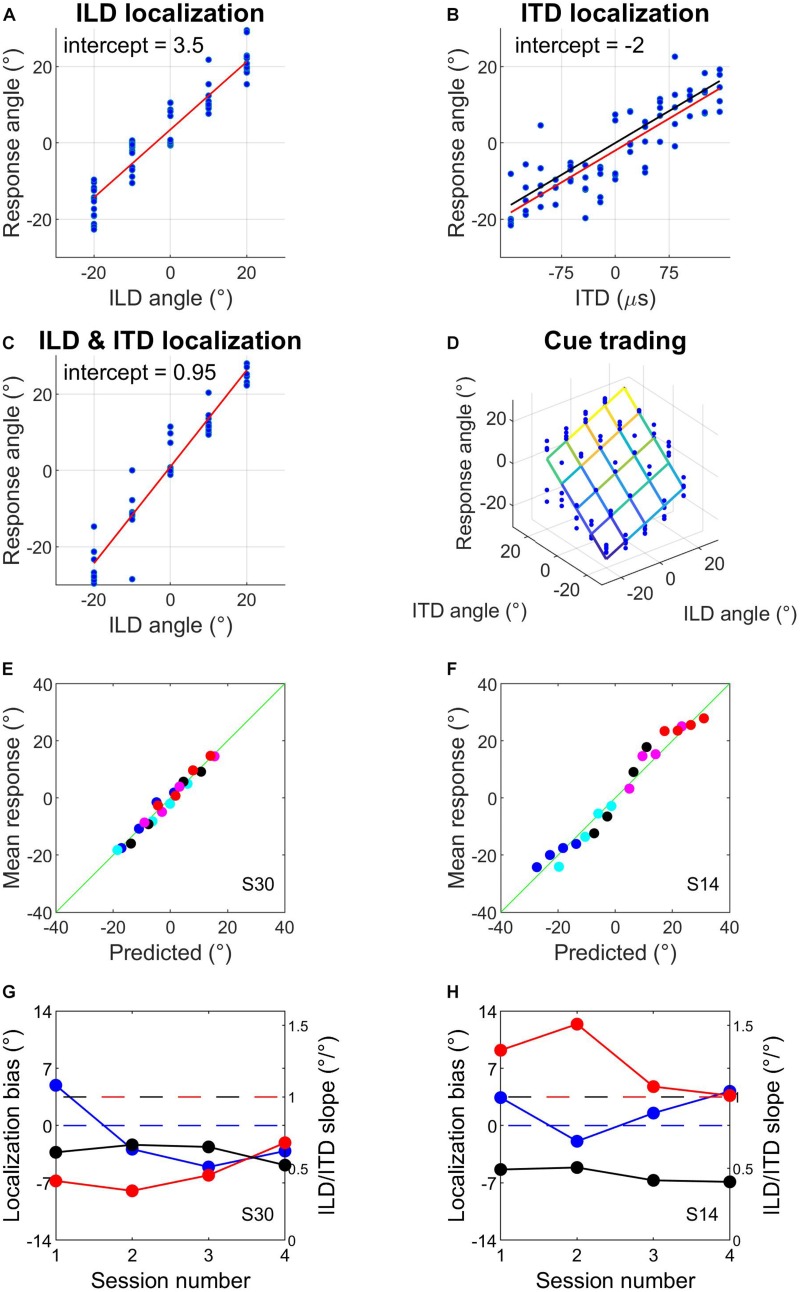
Example data from sound localization tasks and subject screening. **(A–C)** Sound localization with ILDs only **(A)**, ITDs only **(B)**, and ILDs combined with spatially congruent ITD cues **(C)**. Red lines are fits to the data and intercepts indicate subjects’ response biases. In **(B)**, the black line represents the centered function used to match ITDs to response angles (see section Materials and Methods). **(D)** Sound localization in the auditory spatial cue trading task that was carried out before and after audiovisual training. The fit illustrates a two-factor multiple regression of response angle on ILD angle and ITD angle. **(E–H)** Example of well-fitting **(E,G)** and poorly fitting **(F,H)** data when mean pre-test responses from the four audiospatial recalibration experiments were predicted from the multiple regression exemplified in **(D)** (see main text). Symbols of different colors in **(E,F)** indicate different ILD positions; symbols of the same color indicate the different ITD positions that were presented at that ILD. The horizontal blue dashed line in G and H indicates a localization bias of 0° and the red and black dashed lines denote an ILD or ITD slope of 1, respectively.

This first hour involved the same psychophysical tasks, stimuli and binaural cue values as described for day 1. Subjects completed several runs of ILD localization training with visual feedback after each trial, followed by one ILD localization run without visual feedback to check their unsupervised performance with the ILDs (Figure 2A). They then completed several ITD localization runs without visual feedback (Figure 2B) until their performance had stabilized, as indicated by good and reproducible linear fits to the data. We calculated the slope of a linear fit to the ITD localization run with the lowest MSE from the data collected that day. We used this ITD localization slope to calculate ITDs that best matched perceived angles of −20°, −10°, 0°, 10°, and 20°; before doing so we removed the intercept of the fit to avoid introducing systematic biases into the ITD values (Figure 2B). If this was the subject’s first audiospatial recalibration experiment, we checked that sound localization performance with the matched ILD and ITD values was reasonably veridical by having subjects perform a localization run with congruent ILDs and ITDs. This run employed broadband noise (0.5–16 kHz) with reverberation added (Figure 2C). These stimuli therefore provided naturalistic VAS cues, and were used from here onward.

Subjects’ ILD-only and ITD-only localization performance in subsequent experimental sessions tended to stabilize quickly. Therefore, to mitigate subject fatigue before an audiospatial recalibration experiment, we did not reassess localization with spatially congruent ILDs and ITDs in subsequent experiments unless the single-cue training data appeared anomalous due to, for example, the subject attending the session with a cold. On these rare occasions, additional localization testing with visual feedback after each trial was performed and/or the audiospatial recalibration experiment was abandoned and re-run at a later date.

##### Experimental phase – audiospatial recalibration (∼1 h)

In the second hour of each test session, subjects first performed a pre-training auditory localization test (ILD vs. ITD cue trading). This was followed by an AV− or AV+ spatial training task (visual flash detection or audiovisual spatial oddball detection, respectively), and the session concluded with a post-training auditory localization test (ILD vs. ITD cue trading). The tasks were as follows:

##### Cue trading (before and after AV− or AV+ tasks)

This involved measuring sound localization with stimuli containing ILDs and ITDs, and with every combination of the 5 test angles for the two cues. There were therefore 25 different stimuli and each was repeated 5 times. This was performed before and after the AV− and AV+ tasks and subjects were instructed that the stimuli could come from anywhere within the horizontal angular range of the stimuli (i.e., ±20°). From the response data, we estimated the weight of each cue from the slopes of a two-factor multiple regression of response angle on ILD angle and ITD angle (see Figure 2D). The regression intercept corresponded to a subject’s lateral localization bias, i.e., the mean distance of their responses from 0°.

#### Audiospatial Recalibration Tasks

For both the AV− and AV+ tasks, the following four different types of cue statistics were used (Table 2). In each case, subjects were instructed to ignore the auditory stimuli and focus only on the visual aspect of the task [i.e., flash detection (AV−) or spatial oddball detection (AV+)].

(i) *Natural-0; AV− or AV+.* During the auditory stimulus sequence on each trial, ILDs and ITDs were presented from a constant standard location (with the exception of a spatial oddball stimulus), and were always congruent with each other. The standard and oddball locations that were presented across trials covered a range of symmetrical positions around the midline from −20° to + 20°. In the AV+ group the visual stimulus was always congruent with both binaural cues.
(ii) *Random-ITD; AV− or AV+.* ILDs spanned a range of symmetrical positions around the midline and, apart from an ILD oddball in the stimulus sequence, were presented at a constant location during each trial sequence. The ITD cue corresponded to a position up to 20° off the midline that was selected from a uniform random distribution at 10° intervals and changed for each stimulus within a trial sequence. In the AV+ group, the visual stimulus was always congruent with the ILDs.
(iii) *Random-ILD, AV− or AV+.* ITDs spanned a range of symmetrical positions around the midline and, apart from an ITD oddball in the stimulus sequence, were presented at a constant location during each trial sequence. The ILD cue corresponded to a position up to 20° off the midline that was selected from a uniform random distribution at 10° intervals and changed for each stimulus within a trial sequence. In the AV+ group, the visual stimulus was always congruent with the ITDs.
(iv) *Natural-10, AV− or AV+ (ventriloquism aftereffect).* ILDs and ITDs spanned a range of non-symmetrical positions across the midline from *−*10° to +30° (right auditory offset) or *−*30° to +10° (left auditory offset) and were always congruent with each other. In the AV+ group the visual stimulus was presented from a range of symmetrical positions across the midline from *−*20° to + 20°, and was always 10° to the left or right of the auditory stimulus. The offset direction was selected at random, with approximately equal numbers of subjects trained on each side of space.

### Data Analysis

On some trials during pre- and post-test cue-trading runs, hardware failures meant that stimuli were not presented and responses were not recorded. This occurred on 129/32,000 trials (∼0.4%), never more than twice in a cue-trading localization run, and was unrelated to the binaural stimulus combination being presented. These trials were omitted from the dataset before proceeding with the analysis. To confirm that the use of non-individualized HRTFs produced localization responses that could be well described by a simple two-cue linear model, we assessed the fit of the model to the pre-audiospatial recalibration data for each subject. We pooled subjects’ data across their pre-test sessions and fitted the multiple linear regression model given in Equation (1) (Figure 2D).

(1) r⁢e⁢s⁢p⁢o⁢n⁢s⁢e=l⁢o⁢c⁢a⁢l⁢i⁢z⁢a⁢t⁢i⁢o⁢n⁢b⁢i⁢a⁢s+(I⁢L⁢D⁢w⁢e⁢i⁢g⁢h⁢t⋅a⁢n⁢g⁢l⁢e⁢1)+(I⁢T⁢D⁢w⁢e⁢i⁢g⁢h⁢t⋅a⁢n⁢g⁢l⁢e⁢2)

The regression intercept corresponds to the “localization bias,” while the ILD and ITD slopes are considered to be equivalent to the weights of the cues, i.e., their relative contributions to the localization responses. We used the model coefficients to predict subjects’ localization responses for each ILD and ITD cue combination, then we fitted a regression to each subject’s mean predicted scores. We checked each subject’s residuals for autocorrelation using a Durbin–Watson test, and for normality using a Shapiro–Wilk test. Significant results from either test were considered sufficient to flag a subject for exclusion.

To analyze the data from each audiospatial recalibration experiment, we obtained regression intercepts and ILD and ITD slopes from individual pre- and post-training cue-trading test blocks by performing a robust regression using the model given in Equation (1), with an additional interaction term for Session (pre- vs. post-audiospatial recalibration). To characterize group changes in regression coefficients, we fitted the model in Equation (1), again with an interaction term for pre-training vs. post-training, to the entire dataset for each experiment. We determined significant differences between ILD and ITD cue weights before audiospatial recalibration training by examining the confidence intervals of the pre-test coefficients for each cue. We then performed ANOVA on the model coefficients. We further identified general trends in bias and slope changes by regressing post-training biases and slopes on those obtained before audiospatial recalibration after removing outliers using a leverage test (Hoaglin and Welsch, 1978).

To characterize underlying within-subject changes in binaural cue weights, we bootstrapped the mean reciprocal change in ILD and ITD slopes by resampling from the paired within-subject changes in these values with replacement 10,000 times and calculated maxima for the joint distribution of changes in the slopes. To determine how the relative weighting of ILDs and ITDs changed, we derived a “binaural weighting” index (BWI):

(2) $$B\,W\,I=\frac{I\,L\,D\,w\,e\,i\,g\,h\,t}{I\,L\,D\,w\,e\,i\,g\,h\,t+I\,T\,D\,w\,e\,i\,g\,h\,t}-0.5$$

According to this definition, binaural weighting values of −0.5 and 0.5 represent the extreme situations where either ITD or ILD dominate, respectively, and a value of 0 represents equal weighting between the cues. The threshold for determining statistical significance in all analyses was set at *p* < 0.05.

**FIGURE 3:**
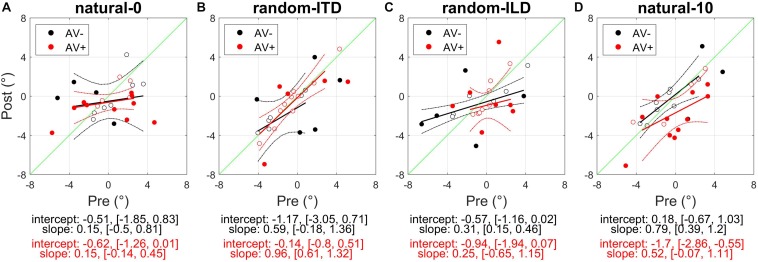
Subjects’ post-recalibration sound localization biases as a function of their pre-training biases across the four experiments. Black and red data points and regression lines respectively indicate subjects trained with spatially-uninformative and spatially-informative visual stimuli. **(A)** natural-0: ILDs and ITDs symmetrical across the midline and congruent. **(B)** random-ITD: ILD constant within each trial sequence and presented from a range of symmetrical locations across the midline, ITD randomized. **(C)** random-ILD: ITD constant within each trial sequence and presented from a range of symmetrical locations across the midline, ILD randomized. **(D)** natural-10: ILDs and ITDs presented from congruent locations offset by an average of 10° from midline and, in the AV+ group, by 10° from the concurrent visual stimulus. Solid symbols indicate significant within-subject changes (see main text). Solid lines are regressions of post-training score on pre-training score after outliers were excluded using a leverage test. Dashed lines are 95% confidence intervals for the fits. Shown below each panel are the fit parameters and their 95% confidence intervals.

**FIGURE 4:**
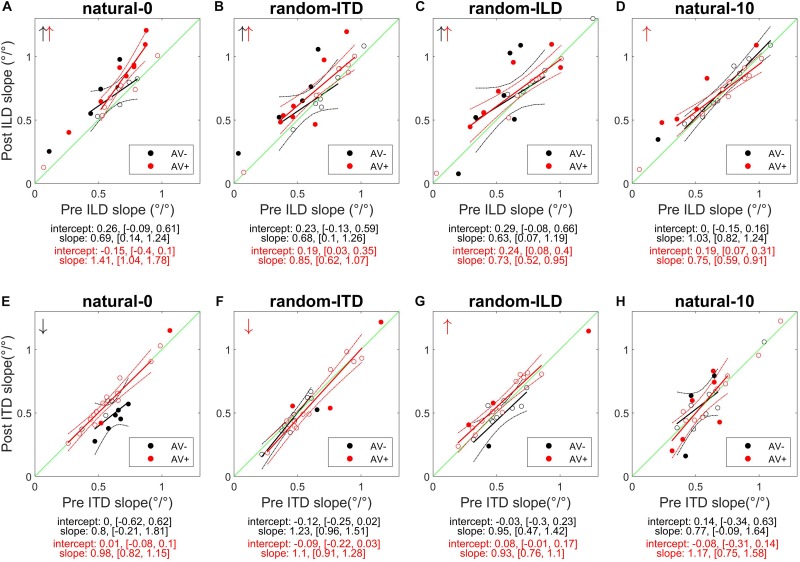
Subjects’ post-recalibration ILD (top row) and ITD (bottom row) cue slopes as a function of pre-training slopes across the four experiments. Black and red data points and regression lines respectively indicate subjects trained with spatially-uninformative and spatially-informative visual stimuli. **(A,E)** natural-0: ILDs and ITDs symmetrical across the midline and congruent. **(B,F)** random-ITD: ILD constant within each trial sequence and presented from a range of symmetrical locations across the midline, ITD randomized. **(C,G)** random-ILD: ITD constant within each trial sequence and presented from a range of symmetrical locations across the midline, ILD randomized. **(D,H)** natural-10: ILDs and ITDs presented from congruent locations offset by an average of 10° from midline and, in the AV+ group, by 10° from the concurrent visual stimulus. Solid symbols indicate significant within-subject changes (see main text). Arrows in the top left of each panel indicate significant group changes according to a linear model (see also Table 4). Solid lines are regressions of post-training score on pre-training score after outliers were excluded using a leverage test. Dashed lines are 95% confidence intervals for the fits. Shown below each panel are the fit parameters and their 95% confidence intervals.

**FIGURE 5:**
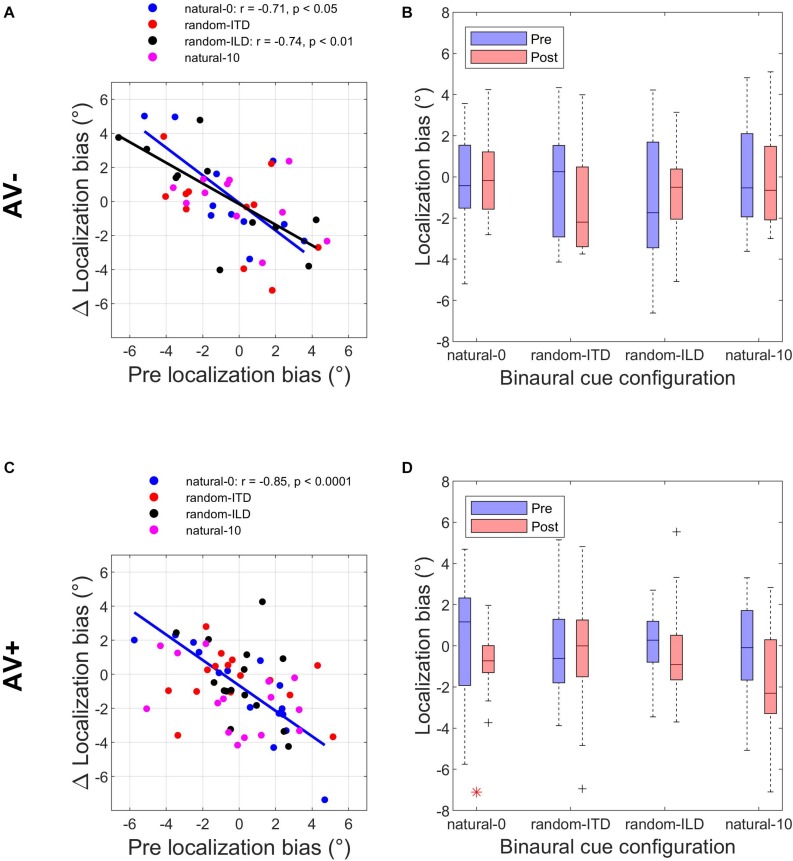
Changes in auditory localization bias. **(A,C)** Change in localization bias regressed on pre-training localization bias after outliers have been disregarded with a test of leverage. Regressions, Pearson’s *r-* and their *p*- values are shown for experiments yielding a significant correlation between pre-training bias magnitude and the magnitude of the bias change induced by audiovisual training with spatially-uninformative (AV−, top row) or spatially-informative (AV+, bottom row) visual stimuli. **(B,D)** Distribution of all subjects’ localization biases before and after training. Horizontal lines in the middle of the boxplots are the medians. Significant reductions in sample variance induced by training are indicated with a red asterisk at the bottom of the panel. Note that in the AV+ group, training with spatially-congruent visual and binaural cues resulted in a narrower range of localization biases across subjects, while training with spatially-offset cues produced the ventriloquism after-effect, with auditory localization responses shifted in the direction of the previously presented visual stimulus.

**FIGURE 6:**
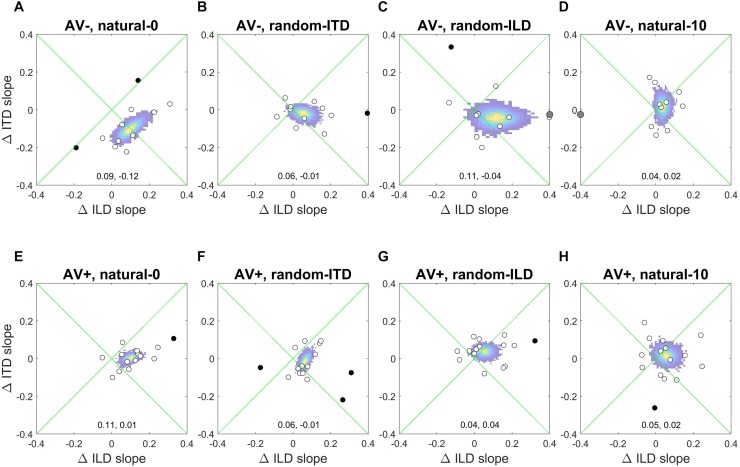
Within-subject ILD and ITD slope changes. **(A–D)**, AV− group. **(E–H)**, AV+ group. White circles are data-points that fell within 2 standard deviations of the mean for both the change in ILD and ITD slope, and were thus included in the bootstrap analysis; black data-points are outliers and gray symbols are data-points falling outside the range of the axes. The colored region indicates the bootstrapped mean of the paired changes in ILD and ITD slopes; the yellow region indicates the most commonly observed values, and the coordinates of the maxima of each distribution are given at the bottom of each panel (ΔILD, ΔITD).

**FIGURE 7:**
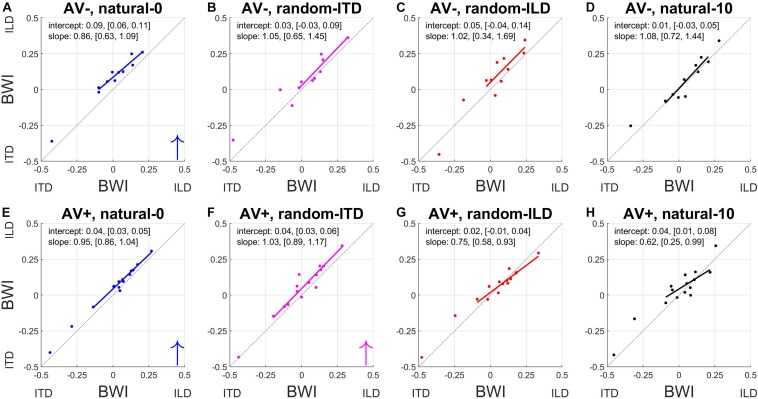
Binaural cue weighting changes for the four audiovisual configurations used in this study. **(A–D)** AV– group. **(E–H)** AV+ group. The solid lines are regressions of pre- training binaural weighting index (BWI) on post-training BWI after outliers have been excluded. Arrows in the bottom right corner indicate the direction of significant group changes in BWI after training (see main text).

The raw data used to generate Figures 2–7 are included in the Supplementary Material.

## Results

### Sound Localization Performance

We first assessed the fit of the model given in Equation (1) to the pre-audiospatial recalibration data for each subject by using the model coefficients to predict subjects’ localization responses. The predictions from the model and the mean angular response are shown for two example subjects in Figures 2E,F. For most subjects, a simple linear cue integration model is a reasonable description of their sound localization performance (Figure 2E). However, the model performed poorly for several subjects (e.g., Figure 2F), with spatially periodic fluctuations in error magnitude possibly caused by systematic cue biases (Figures 2G,H) that arose when one binaural cue made a substantially larger contribution (i.e., was weighted more) than the other to the localization responses. The residual diagnostic tests (see section Materials and Methods) showed that the linear cue integration regression model was a poor fit to the pre-training cue trading data for 6/32 subjects. We therefore excluded the data from those subjects from further analysis.

### Audiospatial Recalibration With Spatially Congruent, Natural Binaural Cues (Natural-0)

In this experiment, subjects performed the visual flash detection (AV− group) or visual spatial oddball (AV+ group) task with ILD and ITD cues that were spatially congruent and distributed evenly around the midline. The AV+ group were also presented with visual stimuli that were spatiotemporally congruent with the binaural cues. Within-subject training-induced changes were assessed by performing robust fits on each subject’s localization data according to the regression model given in Equation (1), with an additional interaction term for test session (pre-training vs. post-training) included. Significant within-subject changes in intercept and slope indicated by these analyses are shown in Figures 3A, 4A,E, respectively. Group-level (AV− or AV+) changes within an experiment were assessed by fitting the same model to the full dataset and performing ANOVA on the model coefficients.

Overall, our results demonstrated that repeated exposure to the binaural cues tended to lead to an improvement in sound localization in the form of a reduction in the magnitude of individual subject’s localization biases, and therefore less variability in the group data (Figure 3A and Table 3). In the AV− group, roughly half of the subjects increased and half decreased their auditory localization biases, although most (7/11) did not show a significant change either way (mean pre-training bias ± SD, −0.42 ± 2.57; post-training −0.07 ± 2.04). This compares with 12/15 subjects becoming less biased (8 significant) when visual and binaural cues were spatiotemporally congruent (AV+; pre-training 0.29 ± 2.83; post-training −0.75 ± 1.49). In contrast to the AV− group, the AV+ group mean localization bias shifted leftwards [ANOVA on group data: AV−, *F*_(__1_, _2744__)_ = 1.38, *p* = 0.24; AV+, *F*_(__1_, _3717__)_ = 15.57, *p* < 0.0001; Table 3]. This trend also approached significance when high-leverage outliers were disregarded, as indicated by the intercept of the regression of post-training on pre-training localization bias (−0.62°: 95% confidence bounds −1.26, 0.01, *t* = −2.19, *p* = 0.053; see Figure 3A).

**TABLE 3:** Summary of descriptive and inferential statistics on subjects’ sound localization biases across the different experiments.

**Visual configuration**	**Experiment**	**Mean ± SD pre-training bias**	**Mean ± SD post-training bias**	**Mean Δ bias (mean Δ abs bias)**	**# subjects with bias decrease (# significant)**	**# subjects with bias increase (# significant)**	**Linear model pre vs. post *p*-value**	**Δ bias vs. pre-training bias**		**Variance ratio (Pitman-Morgan) test results**	
									
								**Pearson’s *r***	***p*-value**	**Variance ratio (pre/post)**	***p*-value**
Spatially and temporally unrelated to auditory stimuli (AV−)	Natural-0	−0.42 ± 2.57	−0.07 ± 2.04	0.36(-0.41)	5 (3)	6 (1)	0.14	–0.71	<0.05^∗^	1.59	0.48
	Random-ITD	−0.67 ± 2.81	−1.17 ± 2.56	−0.50(-0.06)	7 (2)	4 (3)	0.10	–0.56	0.08	1.21	0.74
	Random-ILD	−1.16 ± 3.52	−0.73 ± 2.39	0.42(-1.23)	9 (3)	2 (2)	0.11	–0.74	<0.01^∗^	2.17	0.19
	Natural-10	−0.04 ± 2.60	−0.07 ± 2.48	−0.02(-0.12)	6 (1)	5 (2)	0.64	–0.40	0.22	1.10	0.83

Temporally matched to auditory stimuli and spatially informative (AV+)	Natural-0	0.29 ± 2.83	−0.75 ± 1.49	−1.04(-1.11)	12 (8)	3 (2)	<0.0001^∗^	–0.85	<0.0001^∗^	3.58	<0.05^∗^
	Random-ITD	−0.19 ± 2.63	−0.54 ± 2.86	−0.35(-0.06)	9 (4)	6 (1)	0.20	–0.18	0.53	1.19	0.61
	Random-ILD	0.12 ± 1.69	−0.29 ± 2.30	−0.41(0.48)	5 (5)	10 (2)	0.07	–0.38	0.17	1.86	0.25
	Natural-10	−0.19 ± 2.66	−1.70 ± 2.60	−1.51(0.33)	8 (5)	7 (6)	<0.0001^∗^	–0.40	0.13	1.05	0.90

*Asterisks indicate significant differences according to the tests shown (see section Results).*

For both groups, the magnitude of the change was negatively correlated with subjects’ pre-training biases (AV−, Pearson’s r correlation coefficient = −0.71, *p* < 0.05; AV+, *r* = −0.85, *p* < 0.0001). Thus, the greater the initial bias, the larger the bias reduction after training in both AV− and AV+ groups, although the correlation coefficients suggest this relationship was stronger when training with spatially-informative visual cues was provided in the AV+ group. Our data therefore indicate that although training with spatially congruent visual and auditory cues reduced subjects’ localization biases in most cases, there is some indication that their mean performance converged to a point ∼0.6° to the left of the midline, rather than at the midline itself.

We next examined how the ILD and ITD slopes changed as a result of training on the AV− and AV+ tasks (Figures 4A,E). Prior to training, the ILD and ITD slopes were not significantly different for either the AV− (linear model ILD coefficient and 95% confidence intervals: 0.65 [0.62, 0.69]; ITD 0.66 [0.63, 0.7]), or AV+ (ILD, 0.65 [0.62, 0.67]; ITD 0.61 [0.58, 0.63]) groups (see Table 4). We observed an increase in ILD slopes following training in both groups [5/11 significant subjects in the AV− group, 8/15 in the AV+ group; mean ΔILD slope (and mean percentage change): AV−, 0.08°/° (24%), *F*_(__1_, _2744__)_ = 6.65, *p* < 0.01; AV+, 0.12°/° (23.2%), *F*_(__1_, _3717__)_ = 36.79, *p* < 0.0001; Figure 4A]. In the AV+ group, the slope of the fit to the post- on pre-training ILD slopes is significantly greater than 1 (1.41 [1.04, 1.78]; *t* = 8.78, *p* < 0.0001), indicating that the largest increases in ILD slope occurred in subjects for whom this cue was already weighted strongly. Of the eight AV+ subjects whose ILD slope increases were significant, seven also exhibited significant reductions in their localization biases (compared with just two AV− subjects). There was, however, no correlation between the absolute change in AV+ localization bias and the change in ILD slope (*r* = −0.02, *p* = 0.94).

**TABLE 4:** Summary of descriptive and inferential statistics on ILD and ITD slope across the different experiments.

**Visual configuration**	**Experiment**	**Linear model results: pre-recalibration coefficients [95% CIs]**	**Mean ± SD pre-training slope (^°^/^°^)**	**Mean ± SD post-training slope (^°^/^°^)**	**Mean Δ slope (mean % Δ)**	**# subjects showing decrease (# significant)**	**# subjects showing increase (# significant)**	**Linear model pre vs. post *p*-value**	**Δ binaural weighting index (± SD)**
Spatially and temporally unrelated to auditory stimuli (AV−)	Natural-0	0.65 [0.62, 0.69]	0.65 ± 0.31	0.73 ± 0.25	0.08 (23.97)	2 (1)	9 (5)	<0.01^∗^	0.08 ± 0.03
		0.66 [0.63, 0.70]	0.66 ± 0.27	0.57 ± 0.34	−0.09(-15.93)	8 (6)	3 (1)	<0.001^∗^	
	
	Random-ITD	0.59 [0.56, 0.62]	0.59 ± 0.25	0.68 ± 0.25	0.09 (66.57)	4 (0)	7 (5)	<0.001^∗^	0.04 ± 0.06
		0.58 [0.55, 0.61]	0.57 ± 0.31	0.55 ± 0.31	−0.02(-4.86)	6 (1)	5 (0)	0.44	
	
	Random-ILD	0.66 [0.62, 0.69]	0.66 ± 0.28	0.76 ± 0.33	0.10 (13.7)	2 (2)	9 (4)	<0.01^∗^	0.04 ± 0.08
		0.58 [0.54, 0.61]^∗^	0.59 ± 0.24	0.58 ± 0.35	−0.01(-4.81)	8 (1)	3 (1)	0.67	
	
	Natural-10	0.74 [0.70, 0.77]	0.74 ± 0.35	0.72 ± 0.24	−0.03(5.42)	4 (1)	7 (1)	0.41	0.01 ± 0.05
		0.60 [0.56, 0.63]^∗^	0.58 ± 0.19	0.57 ± 0.24	−0.01(-1.96)	4 (1)	7 (2)	0.46	

Temporally matched to auditory stimuli and spatially informative (AV+)	Natural-0	0.65 [0.62, 0.67]	0.64 ± 0.24	0.76 ± 0.28	0.12 (23.22)	1 (0)	14 (8)	<0.0001^∗^	0.04 ± 0.02
		0.61 [0.58, 0.63]	0.60 ± 0.23	0.61 ± 0.26	0.01 (0.80)	6 (1)	9 (1)	0.91	
	
	Random-ITD	0.65 [0.62, 0.67]	0.65 ± 0.25	0.73 ± 0.28	0.08 (14.03)	2 (1)	13 (6)	<0.0001^∗^	0.04 ± 0.05
		0.65 [0.62, 0.68]	0.64 ± 0.26	0.61 ± 0.29	−0.03(-6.91)	10 (1)	5 (2)	<0.05^∗^	
	
	Random-ILD	0.66 [0.63, 0.68]	0.65 ± 0.28	0.72 ± 0.26	0.07 (27.93)	5 (1)	10 (6)	<0.001^∗^	0.01 ± 0.05
		0.56 [0.53, 0.58]^∗^	0.56 ± 0.26	0.60 ± 0.24	0.04 (10.89)	4 (1)	11 (2)	<0.05^∗^	
	
	Natural-10	0.64 [0.62, 0.67]	0.65 ± 0.28	0.70 ± 0.25	0.05 (19.98)	5 (0)	10 (5)	<0.01^∗^	0.03 ± 0.07
		0.63 [0.61, 0.66]	0.62 ± 0.23	0.62 ± 0.27	−0.001(-1.14)	7 (3)	8 (3)	0.97	

*Where a cell contains two rows of data, the top row is the ILD value and the bottom row is the ITD value. Cells containing asterisks in the third column indicate significant differences between pre-training ILD and ITD slopes according to the confidence intervals of the linear model; asterisks in the ninth column indicate significant changes in slope (see section Results).*

In the AV− group, the ITD slopes changed in a complementary way to the ILD slopes, with a decrease in ITD slope in 8/11 subjects (6 significant) [mean ΔITD slope (and mean percentage change): −0.09°/° (−15.9%), *F*_(__1_, _2744__)_ = 11.92, *p* < 0.001; Figure 4E]. In contrast, ITD slopes in the AV+ group did not change as a result of training [mean ΔITD slope (and mean percentage change): 0.01°/° (0.8%), *F*_(__1_, _3717__)_ = 0.01, *p* = 0.91; Figure 4E].

In summary, repeated exposure to congruent binaural cues reduces auditory localization biases and increases the contribution of ILDs to those responses in many subjects, particularly in the AV+ group. Moreover, the presence of a visual teacher signal in the AV+ group seemed to constrain the concurrent decrease in ITD weight seen in the AV− group.

### Audiospatial Recalibration With Spatially-Consistent ILDs and Randomized ITDs (Random-ITD)

In this experiment, subjects performed the visual flash detection (AV− group) or spatial oddball (AV+ group) tasks with spatially-consistent ILDs, while the ITDs were made unreliable by randomizing them on each stimulus presentation within a trial sequence. We hypothesized that training with these stimuli might result in smaller corrective bias shifts than when the binaural cues were congruent in the natural-0 condition, and that any increase in cue weighting would be restricted to ILDs.

In the AV− group, auditory localization biases increased significantly in 3/11 subjects and decreased in 2, whereas there was essentially no change in the others (Figure 3B and Table 3). As a group, there was no significant change [pre-training mean bias, −0.67 ± 2.81°; post-training, −1.17 ± 2.56°; *F*_(__1_, _2743__)_ = 1.57, *p* = 0.21]. A similar pattern was apparent for the AV+ group, with 4/15 subjects decreasing and 1/15 increasing significantly in bias, and no significant group change [pre-training mean bias, −0.19 ± 2.63°; post-training, −0.54 ± 2.86°; *F*_(__1_, _3719__)_ = 0.95, *p* = 0.33].

Prior to training, regression slopes were the same for ILDs and ITDs in both the AV− (linear model ILD coefficient and 95% confidence intervals, 0.59 [0.56, 0.62]; ITD, 0.58 [0.55, 0.61]) and AV+ (ILD, 0.65 [0.62, 0.67]; ITDs 0.65 [0.62, 0.68]) groups (Table 4). An increase in ILD slope was observed in 7/11 (5 significant) AV− and 13/15 (6 significant) AV+ subjects. Moreover, the mean ΔILD slope (and mean percentage change) were significant in both cases [AV−, 0.09°/° (66.6%), *F*_(__1_, _2743__)_ = 10.9, *p* < 0.001; AV+, 0.08°/° (14%), *F*_(__1_, _3719__)_ = 21.7, *p* < 0.0001], in line with our hypothesis. For both groups, the confidence bounds for the regression slopes shown in Figure 4B encompass 1, indicating that the magnitude of the ILD weight increases was independent of subjects’ starting weights.

Again, as expected for the AV+ group, ITD slopes decreased for 10/15 subjects (1 significant), and we observed a significant overall mean reduction (and mean percentage change) between pre- and post-training values [−0.03°/° (−6.91%), *F*_(__1_, _3719__)_ = 5.48, *p* < 0.05]. The slope of the regression shown in Figure 4F (1.1, [0.91, 1.28]) is not significantly different from 1, implying that the magnitude of the reduction was not related to subjects’ starting ITD slopes. In contrast, the AV− group showed a negligible reduction in ITD slope with no significant group changes [ΔITD slope (and mean percentage change): −0.02°/° (−4.9%); Table 4].

These data indicate that reducing the spatial reliability of ITDs results in smaller localization bias reductions than when congruent binaural cues are available and greater reweighting in favor of ILDs, particularly after training on a task in which ILDs are presented with congruent visual cues.

### Audiospatial Recalibration With Spatially-Consistent ITDs and Randomized ILDs (Random-ILD)

We performed the complementary manipulation in cue reliability to the previous experiment by presenting VAS stimuli with spatially-consistent ITDs and ILDs that were randomized on each stimulus presentation within a trial sequence. Following the results of the random-ITD experiment, we hypothesized that this would also result in smaller corrective bias shifts than when the binaural cues were congruent in the natural-0 condition, but that ITDs rather than ILDs would be upweighted, particularly in the AV+ group.

In the AV− group, auditory localization biases decreased in 9/11 subjects (3 significant) and increased in 2 (both significant) (Figure 3C; see also Table 3). Although the mean group bias shift was not significant [pre-training bias, −1.16 ± 3.52°, post-training bias, −0.73 ± 2.39°; *F*_(__1_, _2743__)_ = 1.65, *p* = 0.2], we observed a negative correlation between the initial localization biases of this group and the direction and magnitude of the change (*r* = −0.74, *p* < 0.01). In other words, initially more biased subjects tended to show the largest bias reductions, which is further illustrated by the low (<1) slope of the fit in Figure 3C (slope [and 95% CIs], 0.31 [0.15, 0.46]). This is therefore consistent with a potential improvement in auditory localization accuracy, even if that was not evident in the bias change in this group as a whole. In the AV+ group, localization biases decreased after training in 5/15 subjects (all significant) and increased in 10 (2 significant), and the group change was not significant [*F*_(__1_, _3721__)_ = 1.89, *p* = 0.17].

In both groups, baseline ILD slopes were significantly higher than ITD slopes (mean and 95% CIs, AV−: ILD, 0.66°/° [0.62, 0.69]; ITD, 0.58°/° [0.54, 0.61]; AV+: ILD, 0.66°/° [0.63, 0.68]; ITD, 0.56°/° [0.53, 0.58]). Our hypotheses about how these weights would change with training were only partly borne out. As expected, ITD weights increased in the AV+ group, where this cue was congruent with the location of the visual stimulus. Increased ITD slopes were seen in 11/15 subjects (2 significant) after training (see Figure 4G), and the group increase was significant [ΔITD slope (and mean percentage change): 0.04°/° (10.9%), *F*_(__1_, _3721__)_ = 6.62, *p* < 0.05]. No overall change was observed in the AV− group [*F*_(__1_, _2743__)_ = 0.18, *p* = 0.67], however, with 8/11 subjects (1 significant) showing a reduction in ITD slope, suggesting that the availability of spatially-informative visual cues is needed to increase the weighting of this cue.

Counter to our expectations, ILD weights increased in 9/11 subjects (4 significant) in the AV− group and in 10/15 subjects (6 significant) in the AV+ group (Figure 4C). In both groups, an increase in slope was observed [ΔILD slope (and mean percentage change): AV−, 0.1°/° (13.7%), *F*_(__1_, _2743__)_ = 10.68, *p* < 0.01; AV+, 0.07°/° (27.9%), *F*_(__1_, _3721__)_ = 11.35, *p* < 0.001]. For the AV+ group the regression slope in Figure 4C is significantly lower than 1 (0.73°/°, [0.52, 0.95]), suggesting that when a spatially relevant visual stimulus was paired with the ILD cue, subjects with the lowest starting slopes exhibited the largest increases in ILD weight.

While the results of this experiment confirm that the slope of the binaural cue whose accuracy is signaled by congruent visual stimuli increases with training, the effect on the randomized cue is more complex and depends on how heavily it is weighted prior to training.

### Audiospatial Recalibration With Spatially Congruent and Asymmetrically Distributed Binaural Cues (Natural-10)

In this experiment, subjects performed the flash detection (AV− group) or audiovisual spatial oddball (AV+ group) task with spatially congruent ILD and ITD cues presented from a range of locations that was biased across the midline by an average of 10°. Subjects in the AV+ group were also presented with concurrent visual stimuli from unbiased locations that were 10° to one side of the binaural cues. The aim of this experiment was therefore to induce a ventriloquism aftereffect (VAE) in the AV+ group and to expose subjects in the AV− group to the same auditory stimuli while they performed a spatially uninformative visual task. Since visual cues highlighting the contextual accuracy of the asymmetric binaural cues were not available, we did not expect to see any cue re-weighting in the AV− group, but anticipated that remapping of auditory space in the VAE might be associated with a down-weighting of the binaural cue that contributed most to baseline localization.

Every subject reported being unaware of the auditory offset when questioned after testing. We first standardized the data for both groups according to the auditory offset direction by reversing the sign of ILD angles, ITD angles and responses for those subjects who had performed this task with the auditory stimuli biased to the left of the midline. In Figure 3D, remapping of post-training localization responses in the direction of the auditory bias is therefore indicated by a shift rightwards in a subject’s multiple regression intercept (i.e., by data-points falling above the green x = y line following training). In the AV+ group, a VAE would be indicated by data points falling below the x = y line.

Figure 3D shows clearly that while there was no change in localization bias for the AV− group [pre-training, −0.04 ± 2.6, post-training, −0.07 ± 2.48; *F*_(__1_, _2743__)_ = 0.16, *p* = 0.69], 12/15 subjects (9 significant) in the AV+ group exhibited post-training biases in the direction of the previously presented visual stimulus [pre-training, −0.19 ± 2.66, post-training, −1.7 ± 2.6; mean shift −1.51°; *F*_(__1_, _3720__)_ = 31.85, *p* < 0.0001]. When we examined only subjects who displayed a significant shift in the direction of the previously presented visual stimulus, the mean VAE size was 2.79 ± 1.06°. The regression slope shown in Figure 3D does not differ significantly from 1 (0.52, [−0.07, 1.11]), indicating that, in general, the shift magnitude was independent of any initial localization bias.

The AV− group baseline cue slopes significantly favored ILD over ITD (linear model coefficients and 95% CIs: ILD, 0.74°/° [0.7, 0.77]; ITD, 0.6°/° [0.56, 0.63]), whereas no difference was seen in pre-training cue weights in the AV+ group (ILD, 0.64°/° [0.62, 0.67]; ITD, 0.63°/° [0.61, 0.66]). After exposure to biased auditory cues in the absence of spatially-informative visual cues, ILD slopes increased in 7 AV− subjects and decreased in the others, with only one significant in either direction [ΔILD slope (and mean percentage change): −0.03°/° (+5.42%), *F*_(__1_, _2743__)_ = 0.68, *p* = 0.41; Figure 4D]. In contrast, ILD slopes increased for 10/15 AV+ subjects (5 significant), and the group increase was also significant [ΔILD slope (and mean percentage change): 0.05°/° (20%), *F*_(__1_, _3720__)_ = 7.2, *p* < 0.01]. However, the confidence bounds [0.59, 0.91] for the slope (0.75) of the regression shown in Figure 4D are less than 1, implying that, during visual recalibration of auditory space, a larger increase in the contribution of this binaural cue was seen in subjects whose ILD slopes were initially low. While a few subjects in both the AV− and AV+ group exhibited significant changes in ITD slope, there were no significant changes at the group level [ΔITD slope (and mean percentage change): AV−, −0.01°/° (−1.96%), *F*_(__1_, _2743__)_ = 0.54, *p* = 0.46; AV+, −0.001°/° (−1.14%), *F*_(__1_, _3720__)_ = 0.001, *p* = 0.97; Figure 4H]. We observed no correlation between the change in AV+ localization bias, i.e., the magnitude of the VAE, and the change in either ILD (*r* = −0.22, *p* = 0.42) or ITD (*r* = 0.3, *p* = 0.27) slope, and two subjects who did not exhibit significant changes in the slopes of either cue nonetheless displayed significant shifts in localization bias. Thus, while this experiment confirms our previous findings in demonstrating that ILD is the cue most likely to be up-weighted as a result of audiovisual training, the visually-induced remapping of auditory space was not always accompanied by changes in cue weights.

### Reduction in Inter-Subject Range of Sound Localization Bias

The experiments described above demonstrated that when one or both binaural cues were aligned with the visual stimulus (AV+ group: natural-0, random-ITD, and random-ILD), only the natural-0 and random-ILD configurations produced significant auditory localization bias reductions in a substantial number of subjects. Furthermore, the flat slopes for the AV+ natural-0, AV− natural-0, and AV− random-ILD fits in Figures 3A,C imply that where bias reductions were observed, larger reductions occurred for those subjects who were most biased to begin with. We confirmed this by fitting the robust regressions shown in Figures 5A,C (fits are shown only for significant correlations between the change in bias and its starting value). Although the slopes are similar for the three significant fits (AV−, natural-0: −0.81 [−1.46, −0.16]; AV−, random-ILD: −0.61 [−0.96, −0.25]; AV+, natural-0: −0.75 [−1.06, −0.43]), the reduction in mean localization bias was more complete when binaural cues were congruent in the natural-0 configuration and presented with spatially-informative visual cues (Pearson’s *r*: AV−, natural-0: −0.71; AV−, random-ILD: −0.74; AV+, natural-0: −0.85).

We reasoned that group-level mean reductions in localization bias would also be associated with group-level reductions in the spread of individual subject biases. To test whether any of the stimulus configurations used in the AV− and AV+ groups led to significant reductions in bias range, we compared the distributions of these values before and after audiospatial recalibration by subjecting them to paired-variance ratio tests. The pre- and post-training localization bias distributions are shown in Figures 5B,D. A significant reduction in variance was observed only in the AV+, natural-0 experiment, where both the ILD and ITD cues were presented with a spatially congruent visual stimulus, according to both a parametric (Pitman-Morgan, pre:post variance ratio = 3.58, *p* < 0.05) and non-parametric (Grambsch, *z* = 2.48, *p* < 0.05) paired variance ratio test.

Thus, based on reductions in mean bias and in the range of responses across subjects, it appears that training with spatiotemporally congruent audiovisual cues produces a larger improvement in sound localization at the group level than exposure to the same auditory stimuli that are not coupled to spatially-informative visual stimuli. By contrast, while the VAE condition induced an adaptive shift in auditory bias, the range of the subjects’ responses across this group was unchanged, implying that visually-guided remapping of auditory space is not necessarily associated with less inter-subject variation.

### Changes in Binaural Cue Contributions to Localization Responses

Figure 6 shows the absolute changes in ILD and ITD slopes for each subject in each experiment, as well as the bootstrapped coordinates of the mean within-subjects ILD and ITD change (shown at the bottom of each panel) to illustrate the underlying pattern of slope changes. Where cue weights did change, they tended to involve simply an increase in the contribution of ILDs to the localization responses. Note, however, the large reciprocal alteration in ILD and ITD slopes in the AV− group when both binaural cues were congruent with each other and distributed symmetrically around the midline (natural-0; Figure 6A). Similarly, the underlying changes in the AV+ random-ILD experiment (Figure 6G) appear to be correlated. These data therefore suggest that the auditory system can adjust the representation of different cues flexibly and cooperatively or independently, depending on the context in which the cues are presented.

### Changes in Relative Weighting of Binaural Cues

To understand how these changes in ILD and ITD slopes reflected changes in the relative weighting of each cue, we calculated the BWI for each subject (see section Materials and Methods), for which values of −0.5 and 0.5 represent the extreme situations where either ITD or ILD dominate, respectively, and a value of 0 represents equal weighting between the cues. The pre- and post-recalibration BWI for each experiment is shown in Figure 7.

The natural-0 experiment in the AV− group yielded a slightly negative mean BWI before audiospatial recalibration (i.e., on average, the ITD was weighted higher; mean ± SD, −0.006 ± 0.17), but for the other seven experiments the baseline BWI was positive, indicating that localization responses were more dependent on ILDs. Accordingly, with all subjects’ pre-training scores combined, we observed significantly higher ILD than ITD weights (BWI mean ± SD = 0.02 ± 0.18, sign-rank test, *p* < 0.05). Increases in ILD weighting occurred in the natural-0 experiment (Figure 7) whether spatially-informative visual stimuli were presented or not (sign-rank tests, mean ± SD: AV−, natural-0: 0.08 ± 0.03, *p* < 0.0001; AV+, natural-0: 0.04 ± 0.02, *p* < 0.0001), but we also saw an increase in ILD weight when spatially-relevant visual stimuli were congruent with the ILD and the ITD position was randomized (AV+, random-ITD: 0.04 ± 0.05, *p* < 0.01). A significant change in BWI was not observed in the other experiments, although small increases in ILD weights were observed as a result of training (Table 4, final column and Figures 7B,C).

Given that there were differences between the binaural weights at baseline in our sample population, it was important to establish whether the experience-induced reweighting depended on the pre-training BWI values. The way the BWI changed in the natural-0 and random-ITD experiments was independent of the baseline relative weighting of these cues, suggesting that the magnitude of the change in weighting in favor of ILDs was consistent irrespective of the starting weight (indicated by the pre vs. post BWI regression slopes in Figures 7A,E,F). Although we found no overall change in binaural weighting in the other experiments, the regression slopes indicate that when visual cues signaled that ILDs were unreliable (AV+, random-ILD) or that both cues were inaccurate (AV+, natural-10), there was a tendency for the weighting of whichever cue contributed less at baseline to increase as a result of training (Figures 7G,H).

**FIGURE 8:**
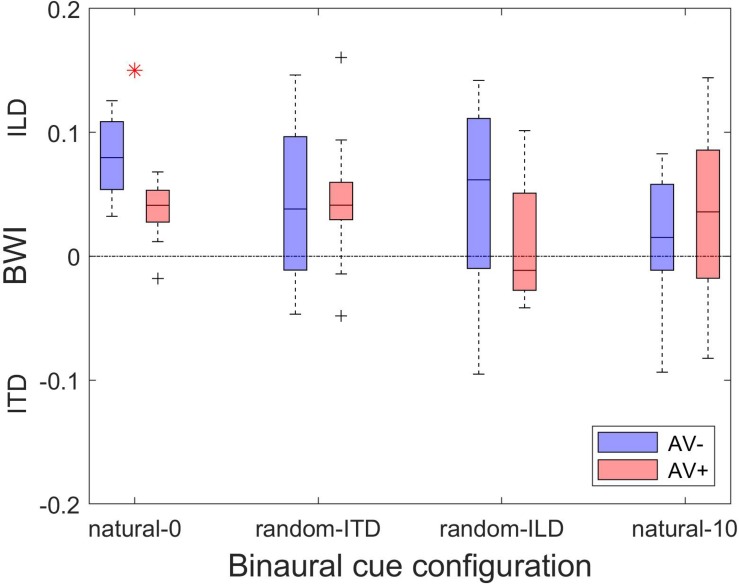
Changes in binaural cue weighting for the AV− and AV+ groups. Positive values indicate re-weighting favoring the ILD cue, negative values indicate an increase in weighting favoring the ITD cue. Red asterisks indicate significant differences in cue weighting changes between the AV− and AV+ groups according to a two-sample *t*-test.

Finally, comparison of the magnitude of the change in BWI between the AV+ and AV− groups demonstrated that the only difference was seen in the natural-0 experiment, in which, surprisingly, ILD was up-weighted to a greater extent in the AV− group than in the AV+ group [two-sample *t*-tests: natural-0, *t*_(__24__)_ = 4.07, *p* < 0.0001; random-ITD, *t*_(__24__)_ = −0.04, *p* = 0.97; random-ILD, *t*_(__24__)_ = 1.4, *p* = 0.17; natural-10, *t*_(__24__)_ = −0.75, *p* = 0.46; Figure 8].

## Discussion

In this study, we measured changes in the weighting of different auditory spatial cues when humans localize broadband sounds filtered through non-individualized HRTFs. We used VAS stimuli to manipulate the relative reliability of ITDs and frequency-dependent ILDs in different ways during ∼1 h long training sessions. This was done by randomizing one cue within each sound sequence while keeping the other constant, in addition to presenting spatially-congruent values of both cues corresponding to a range of virtual sound directions either symmetrically around the midline or offset by 10° to one side. One group of subjects (AV−) performed a spatially-uninformative visual detection task during exposure to these auditory stimuli, whereas the other group (AV+) performed a visual oddball task with visual cues that were either spatiotemporally congruent with or displaced by 10° from the spatially consistent binaural cue(s). The purpose of the visual stimulus in the AV+ group was therefore to provide an additional contextual signal to influence the perceived accuracy of the auditory spatial cue with which it was paired.

Our results show that a visual “teacher” signal induces corrective changes in auditory localization (characterized by individual reductions in bias, and a narrower range of biases across subjects) only when it is spatially congruent with all available sound localization cues. However, even in the absence of spatially-informative visual cues, some subjects displayed a reduction in localization bias when binaural cues were consistent with each other, or when they were exposed to spatially-consistent ITDs in conjunction with ILDs that were randomized on each stimulus presentation within a trial sequence. Furthermore, in line with other studies of remapping of auditory space by vision (Radeau and Bertelson, 1974; Recanzone, 1998), we found that presenting congruent binaural cues that were consistently offset by 10° from a visual stimulus (VAE) induced a ventriloquism aftereffect of 1.5–2.8°, but no changes in the variability in bias across subjects.

These shifts in localization bias were accompanied by context-dependent changes in the weights of the auditory cues (defined as the slopes of regressions of response on ILD and ITD angle), leading to significant cue re-weighting in some cases. This was most clearly observed when the binaural cues were congruent, irrespective of whether visual cues were spatially informative or not, and involved an increase in the contribution of ILDs to the auditory localization responses. However, ILDs were also up-weighted when this auditory cue was spatially congruent with vision and ITDs randomized on each trial. Thus, changes in auditory localization bias and cue weighting can be induced by exposure to a range of virtual sound directions while subjects perform an irrelevant visual task, with more extensive changes resulting from training on a spatially-informative visual task.

### Plasticity in Auditory Spatial Processing

Because the VAS stimuli were presented using non-individualized HRTFs, we would expect the listeners’ localization judgments to be less accurate than if the stimuli had been based on measurements from their own ears (Wenzel et al., 1993; Middlebrooks, 1999; Mrsic-Flogel et al., 2001). Other studies have shown that human listeners can, at least to some extent, learn to accommodate the inappropriate auditory spatial cues provided by non-individualized HRTFs (Zahorik et al., 2006; Mendonça et al., 2013; Berger et al., 2018; Stitt et al., 2019). Although the participants in our study were told to ignore the sounds, some subjects in the AV− group, who did not have the benefit of spatially informative visual cues, showed a reduction in localization bias, particularly if the bias was relatively high prior to training. This was the case when the binaural cues were spatially congruent or when the ILD cue was randomized. Furthermore, changes in binaural cue weighting were observed in this group. Thus, even in the absence of spatiotemporally aligned visual cues, passive exposure to the sounds was sufficient to induce some changes in auditory localization performance. This is consistent with other studies demonstrating that sensitivity to binaural cues can be recalibrated by modifying the statistics of sound stimulation (Dahmen et al., 2010; Maier et al., 2012; Stange et al., 2013).

### Vision Shapes Auditory Localization

Our experiments provide further evidence for the marked influence of vision on auditory localization (e.g., Radeau and Bertelson, 1974; Recanzone, 1998; Alais and Burr, 2004; Bolognini et al., 2007; Tabry et al., 2013; Hammond-Kenny et al., 2017). We found that training on a visual spatial task that was accompanied by auditory stimuli can enhance or shift post-training auditory localization responses depending on the spatial relationship between the visual and auditory cues. Although the auditory system can learn to accommodate impoverished or altered auditory spatial cues over a period of days to weeks in the absence of vision (Kacelnik et al., 2006; Carlile and Blackman, 2014), the addition of visual spatial cues can promote such adaptation (Strelnikov et al., 2011; Isaiah et al., 2014). Our data show that remapping of auditory space (i.e., shifts in localization bias) and re-weighting of auditory localization cues are apparent after less than 1 h of training. That rapid reductions in localization bias can be achieved even when using another person’s ears confirms other research demonstrating that brief exposure to audiovisual stimuli leads to an improvement in auditory localization with non-individualized HRTFs (Berger et al., 2018). Indeed, remapping of auditory space can manifest after a single exposure to an audiovisual disparity (Wozny and Shams, 2011b; Bruns and Röder, 2015).

When training was provided with spatiotemporally congruent visual and auditory cues, we found the biases tended to correct to a point just to the left of the midline rather than at the midline itself. This may find an explanation in recent studies of pseudoneglect that demonstrate opposite spatial attentional biases for visual and auditory stimuli in healthy listeners, such that performance in spatial attention tasks is characterized by a leftward bias for visual stimuli (Loftus and Nicholls, 2012; Thomas et al., 2014, 2017) and a rightward bias for stimuli presented to the auditory modality (Sosa et al., 2010, 2011). These biases have been postulated to result from hemispheric asymmetries in controlling the deployment of spatial attention. Reports of a greater potency of visual capture of auditory cues in the left vs. the right visual hemifield (Sosa et al., 2011) are also consistent with the tendency of several of our subjects to show a small leftward bias in sound localization following training with spatially congruent audiovisual cues.

### Changes in Binaural Cue Weighting

Although we did not measure localization accuracy for the cues in isolation and therefore cannot determine their relative contributions to the changes in bias, the subjects in the AV− group who showed bias reductions had access to spatially consistent ITDs (i.e., the natural-0 and random-ILD conditions). When spatially-informative visual cues were available in the AV+ subjects, group-level changes in bias were observed when ITDs and ILDs were spatially consistent and congruent. While this result provides some indication that the spatial reliability of ITDs may be important in determining whether adaptive changes in localization occur or not, our subjects generally weighted ILDs higher than ITDs when localizing broadband sounds.

This contrasts with previous studies in which the relative contribution of different spatial cues has been assessed by investigating the effects of manipulating each cue on localization responses (e.g., Wightman and Kistler, 1992; Macpherson and Middlebrooks, 2002). However, our results are consistent with a previous observation of more salient ILDs when binaural cues are presented over headphones in multisensory experiments (Kumpik et al., 2014). A possible explanation for this may be that the reverberation we applied to help subjects externalize the auditory stimuli was temporally congruent with the stimulus onset in the left and right channels. This may have smeared the ITDs present in the onsets, offsets and ongoing amplitude envelopes of our stimuli (Shinn-Cunningham and Kawakyu, 2003; Rakerd and Hartmann, 2010), leading to those cues becoming less reliable. Furthermore, in the hour before every experimental session (sessions 2–5) subjects received substantial ILD localization training, with visual feedback after each trial, to ensure reasonably veridical localization with the ILDs provided by their selected non-individualized HRTF.

We found that ILDs were upweighted in both AV− and AV+ recalibration tasks when the two binaural cues were spatially congruent. If spatially-informative visual cues were available, an increase in the relative weighting of ILDs was also observed when they matched the visual cues and the ITDs were randomized. This implies that the direction of cue re-weighting followed the relative weights measured prior to training, which, in these experiments, were generally higher for ILD, as well as the availability of congruent visual cues. Thus, in mapping physical sound localization cues to external spatial positions the brain dynamically takes account of the contextual reliability of all the available information (Rohe and Noppeney, 2015) and adjusts the spatial weighting of the of the cues accordingly.

Even where these changes were insufficient for cue re-weighting to take place, we often found that increases in ILD weights occurred with training (which, surprisingly, included the random-ILD experiment in which ITDs were spatially consistent and congruent with vision, while ILDs were randomized within each sound sequence). Subjects reported verbally that in this experiment they found the randomized auditory cue to be highly salient and difficult to ignore; for at least the first 2–3 spatial oddball (AV+) or flash-detection (AV−) training blocks, they made involuntary eye movements toward the randomized position of the ILD cue, likely reflecting sound-driven orienting responses that help to select relevant stimuli for spatial attention amongst competing distractors (Krauzlis et al., 2013; Hartmann et al., 2016). This suggests that cue-specific effects of exogenous auditory attention also need to be considered when investigating how the brain weights different sources of spatial information.

Our finding that the relative weighting of auditory localization cues can be changed by experience is consistent with previous work demonstrating that the auditory system can adapt to altered auditory inputs resulting from occlusion of one ear by reweighting the available localization cues. In particular, intact spectral localization cues are up-weighted at the expense of binaural cues that now provide conflicting or unreliable information (Kacelnik et al., 2006; Van Wanrooij and Van Opstal, 2007; Kumpik et al., 2010; Keating et al., 2013, 2016). However, remapping of the binaural cues has also been observed (Keating et al., 2015, 2016; Trapeau and Schönwiesner, 2015), with re-weighting and remapping of different sound localization cues appearing to take place independently (Keating et al., 2016). Similarly, our results show that shifts in localization bias can occur alongside, but are not necessarily associated with, cue re-weighting.

Auditory localization cues appear to be re-weighted following monaural occlusion according to how reliable or accurate they are (Kacelnik et al., 2006; Van Wanrooij and Van Opstal, 2007; Kumpik et al., 2010). This is consistent with our finding that ILDs were upweighted when they were aligned with visual cues, but not when they were randomized relative to the ITD. It also fits well with studies that model optimal cue integration using the ideal-observer (e.g., Alais and Burr, 2004), but there is emerging evidence that sensory cue integration, let alone recalibration, may not follow principles of optimality (Arnold et al., 2019; Meijer et al., 2019). Interestingly, where cue weighting did change in our study, we observed a consistent shift irrespective of the pre-training binaural weighting index. This lends support to work suggesting that the degree of sensory cue recalibration may follow a fixed-ratio model, where the relative amount of adaptation in the cues is the same and independent of their relative reliability (Zaidel et al., 2011). We believe this to be the first direct demonstration of training-induced binaural cue re-weighting under normal hearing conditions.

### Neurophysiological Basis for Plasticity

Studies in patients with brain lesions suggest that short-term auditory adaptation to spatially congruent and incongruent audiovisual stimuli is subserved by distinct neural circuits (Passamonti et al., 2009; Bertini et al., 2016), with congruent stimuli activating a circuit involving the superior colliculus (SC) and extrastriate visual cortex, resulting in a reduction in position-specific auditory localization errors. Conversely, the geniculostriate pathway has been implicated in a generalized recalibration of auditory space for incongruent stimuli, probably as a result of a direct biasing of activity in auditory cortical areas, such as the planum temporale, by visual signals (Bonath et al., 2007, 2014; Bertini et al., 2010). In line with this, recent work (Zierul et al., 2017) points to changes in the balance of activity and in the degree of interaction between the left and right primary auditory cortices, associated with an increase of connectivity between temporal and parietal regions along the auditory “where” pathway, as a likely neural correlate of the VAE. That VAE-associated activations have been shown to occur around 160 ms earlier than those associated with the ventriloquism effect (e.g., Bonath et al., 2007; Bruns et al., 2011) supports the notion that the VAE arises from a modulation of early auditory perceptual processing.

Both re-weighting of spectral cues (Keating et al., 2013) and adaptive shifts in sensitivity to ILDs (Keating et al., 2015) take place in the primary auditory cortex of ferrets raised with one ear occluded, with largely separate populations of neurons undergoing each form of plasticity (Keating et al., 2016). Similarly, adaptation to modified ITDs in adult humans is associated with changes in auditory cortical activity (Trapeau and Schönwiesner, 2015). Consequently, the cue weight changes and bias corrections observed after training with spatially-uninformative visual cues could be mediated at the level of auditory cortex. Furthermore, under normal hearing conditions, spatial processing in the auditory cortex is enhanced by congruent visual inputs (Bizley and King, 2008) and by performance of a visual task requiring subjects to direct their attention to the left or right (Salminen et al., 2013).

While consistent with the possibility that the auditory cortex is involved in the recalibration of auditory cue sensitivity, adaptive changes in the coding of binaural cues that are sufficient to account for the effects of sound statistics on spatial perception have been demonstrated at subcortical levels (Dahmen et al., 2010; Maier et al., 2012; Stange et al., 2013). Moreover, vision can influence subcortical auditory spatial processing, both in the SC itself and potentially in the inferior colliculus, which is reciprocally connected with the SC (Doubell et al., 2000; Stitt et al., 2015), and where neurons are sensitive to changes in the alignment of sound localization cues (Slee and Young, 2014). How cortical and midbrain circuits interact to bring about visually-induced shifts in perceived sound-source location and changes in the underlying auditory localization cue weights remains to be determined.

## Conclusion

In summary, our results show that training human participants on visual flash detection or visual spatial oddball detection tasks that were accompanied by broadband sounds can induce rapid changes in auditory localization accuracy alongside, or independently of, changes in the weighting of the cues that provide the basis for spatial hearing. While changes in localization bias and cue weighting can take place in the absence of spatially-informative visual stimuli, larger effects, particularly on the accuracy of sound localization, were observed when visual spatial cues were concurrently available. These findings illustrate the remarkable plasticity of the auditory system and have implications for the design of auditory training schemes aimed at enhancing the sound localization abilities of hearing-impaired listeners, as well as for achieving optimal human interaction with auditory displays in real and virtual environments.

## Data Availability Statement

The raw data supporting the conclusions of this manuscript will be made available by the authors, without undue reservation, to any qualified researcher.

## Ethics Statement

This study was carried out in accordance with the recommendations of the Medical Sciences Inter-Divisional Research Ethics Committee of the University of Oxford with written informed consent from all subjects. All subjects gave written informed consent in accordance with the Declaration of Helsinki. The protocol was approved by the Medical Sciences Inter-Divisional Research Ethics Committee of the University of Oxford (study R52936).

## Author Contributions

DK, JS, and AK conceived and designed the study and wrote the manuscript. DK and CC carried out the experiments and analyzed the data.

## Conflict of Interest

The authors declare that the research was conducted in the absence of any commercial or financial relationships that could be construed as a potential conflict of interest.

## Supplementary Material

TABLE S1 Read me file and raw data for Figures 2–7.
